# Mitochondrial Toxicogenomics for Antiretroviral Management: HIV Post-exposure Prophylaxis in Uninfected Patients

**DOI:** 10.3389/fgene.2020.00497

**Published:** 2020-05-26

**Authors:** Maria Bañó, Constanza Morén, Sergio Barroso, Diana Luz Juárez, Mariona Guitart-Mampel, Ingrid González-Casacuberta, Judith Canto-Santos, Ester Lozano, Agathe León, Enric Pedrol, Òscar Miró, Ester Tobías, Josep Mallolas, Jhon F. Rojas, Francesc Cardellach, Esteban Martínez, Gloria Garrabou

**Affiliations:** ^1^Muscle Research and Mitochondrial Function Laboratory, Cellex-August Pi i Sunyer Biomedical Research Institute (IDIBAPS), Faculty of Medicine and Health Science-University of Barcelona, Internal Medicine Department, Hospital Clínic of Barcelona, Barcelona, Spain; ^2^U722 CIBERER, Barcelona, Spain; ^3^Infectious Disease Department, Hospital Clinic of Barcelona, Barcelona, Spain; ^4^Internal Medicine Department, Hospital de Viladecans, Barcelona, Spain; ^5^Emergency Department, Hospital Clinic of Barcelona, Barcelona, Spain

**Keywords:** ART, HIV, mitochondria, mtDNA, PEP

## Abstract

**Background:** Mitochondrial genome has been used across multiple fields in research, diagnosis, and toxicogenomics. Several compounds damage mitochondrial DNA (mtDNA), including biological and therapeutic agents like the human immunodeficiency virus (HIV) but also its antiretroviral treatment, leading to adverse clinical manifestations. HIV-infected and treated patients may show impaired mitochondrial and metabolic profile, but specific contribution of viral or treatment toxicity remains elusive. The evaluation of HIV consequences without treatment interference has been performed in naïve (non-treated) patients, but assessment of treatment toxicity without viral interference is usually restricted to *in vitro* assays.

**Objective:** The objective of the present study is to determine whether antiretroviral treatment without HIV interference can lead to mtDNA disturbances. We studied clinical, mitochondrial, and metabolic toxicity in non-infected healthy patients who received HIV post-exposure prophylaxis (PEP) to prevent further infection. We assessed two different PEP regimens according to their composition to ascertain if they were the cause of tolerability issues and derived toxicity.

**Methods:** We analyzed reasons for PEP discontinuation and main secondary effects of treatment withdrawal, mtDNA content from peripheral blood mononuclear cells and metabolic profile, before and after 28 days of PEP, in 23 patients classified depending on PEP composition: one protease inhibitor (PI) plus Zidovudine/Lamivudine (PI plus AZT + 3TC; *n* = 9) or PI plus Tenofovir/Emtricitabine (PI plus TDF + FTC; *n* = 14).

**Results:** Zidovudine-containing-regimens showed an increased risk for drug discontinuation (RR = 9.33; 95% CI = 1.34–65.23) due to adverse effects of medication related to gastrointestinal complications. In the absence of metabolic disturbances, 4-week PEP containing PI plus AZT + 3TC led to higher mitochondrial toxicity (−17.9 ± 25.8 decrease in mtDNA/nDNA levels) than PI plus TDF + FTC (which increased by 43.2 ± 24.3 units mtDNA/nDNA; *p* < 0.05 between groups). MtDNA changes showed a significant and negative correlation with baseline alanine transaminase levels (*p* < 0.05), suggesting that a proper hepatic function may protect from antiretroviral toxicity.

**Conclusions:** In absence of HIV infection, preventive short antiretroviral treatment can cause secondary effects responsible for treatment discontinuation and subclinical mitochondrial damage, especially pyrimidine analogs such as AZT, which still rank as the alternative option and first choice in certain cohorts for PEP. Forthcoming efforts should be focused on launching new strategies with safer clinical and mitotoxic profile.

**Graphical Abstract:**
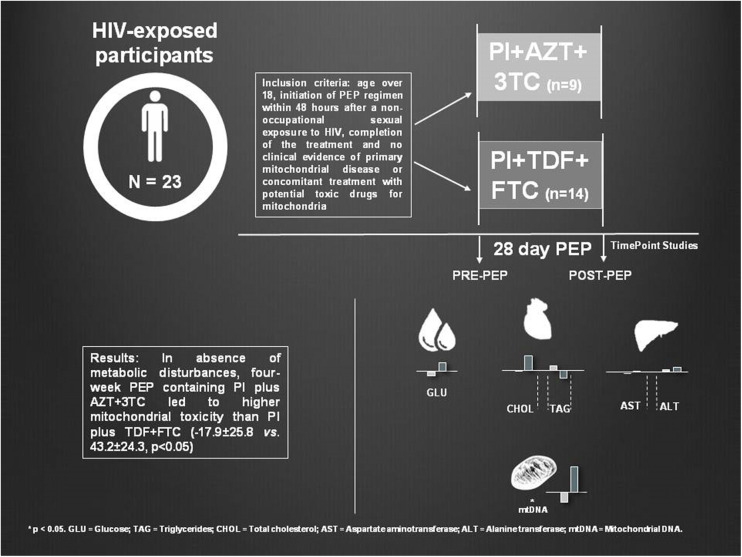
Post-exposure prophylaxis (PEP) myotoxicity.

## Highlights

- PEP regimens are metabolically safe.
- PEP antiretrovirals, in absence of HIV infection, is able to induce mitochondrial toxicity. Currently recommended PEP regimens show less mitochondrial toxicity than the old ones containing pyrimidine analogs such as AZT and 3TC. However, AZT is still used in certain clinical and geographical settings.
- AZT-containing regimens showed a higher risk of drug discontinuation.
- Reduction of mitochondrial toxicity of PEP regimens may improve tolerability and toxicity issues.
- Current and forthcoming efforts to elaborate global policy guidelines should consider mitochondrial toxicity of PEP as an important issue for compliance and patient care.
- PEP-treated patients convey an outstanding opportunity to assess antiretrovirals toxicity *in vivo*.
- mtDNA is confirmed as the gold standard for mitochondrial toxicogenomics in antiretroviral management.

## Introduction

Mitochondria are the energy and heat power plants of the cell (Nunnari and Suomalainen, 2012). These organelles harbor their own enzymatic machinery and all the structures required for the transcription and translation of their own genome, the mitochondrial DNA (mtDNA) (Anderson et al., 1981). Any disbalance in mitohormesis can lead to disease (Boczonadi and Horvath, 2014; Suomalainen and Battersby, 2017; Eisner et al., 2018). Thus, genetic but also epigenetic modifications in the mitochondria can be associated with a variety of metabolic modifications described in a multitude of adverse conditions, including cancer and neurodegenerative diseases as well as biological processes as aging (Moosavi and Motevalizadeh Ardekani, 2016; Weinhouse, 2017; Raimundo and Krisko, 2019). Moreover, the study of mitochondrial genome has been used in fields as population genetics, forensic science, clinical diagnosis, and toxicogenomics (Castro Antönia and Ramon, 1998; Budowle et al., 2003; Chinnery and Hudson, 2013).

A multitude of evidence demonstrates that any toxic agent interfering at genetic or epigenetic level with mtDNA can potentially disrupt mitochondrial function and induce metabolic disturbances and their associated clinical consequences (Alston et al., 2017; Matilainen et al., 2017).

Historically, several compounds have been found to damage mtDNA, including biological and therapeutic agents. This is the case with both the human immunodeficiency virus (HIV) and its antiretroviral treatment (ART) (Miro et al., 2005; Margolis et al., 2014; Smith et al., 2017). HIV induces mitochondrial-driven apoptosis, indirectly reducing mtDNA content (Mbita et al., 2014). Moreover, ART—especially nucleoside reverse transcriptase inhibitors analogs (NRTIs)—interferes with the replication of the viral genome, but secondarily by off-targeting the replication of the mtDNA through the inhibition of mtDNA-polymerase-γ (Brinkman et al., 1999; Kakuda, 2000; Nolan and Mallal, 2004; Feeney et al., 2010; Zhang et al., 2014). This process subsequently triggers mtDNA depletion and derived mitochondrial and cell dysfunction, which has been postulated as the basis for associated clinical toxicity (Carr and Cooper, 2000; Lim and Copeland, 2001).

Zidovudine (AZT), the prototype NRTI class drug, is a pyrimidine analog linked to long-term secondary effects. Included in this group, and combined with AZT is Lamivudine (3TC), with lesser harmful effects (World Health Organization, 2018). Both of these drugs in long-term usage result in different secondary effects such as myelosuppression or myopathy, among others (Kinloch-de Loës et al., 1995; Quercia et al., 2018).

To avoid these adverse effects, other NRTIs such as Tenofovir (TDF) emerged (Scherzer et al., 2012; Margolis et al., 2014; Yap et al., 2019). TDF in combination with Emtricitabine (FTC), another NRTI, constitutes the main 2xNRTI combination included in the ART proposed by the main institutions (Centers for Disease Control Prevention, 2016; Battegary et al., 2018; World Health Organization, 2018). FTC is a dideoxycytidine analog with a structure similar to 3TC, being considered as bioequivalent drugs even from the toxic point of view (Birkus et al., 2002; Margolis et al., 2014).

*In vitro* studies have ranked the potencies of these four NRTIs to inhibit mtDNA synthesis as follows: Zidovudine > Lamivudine = Emtricitabine = Tenofovir (Kakuda, 2000; Birkus et al., 2002). Therefore, mtDNA quantification has been established as the hallmark of antiretroviral toxicity and the gold standard for assessing mitochondrial toxicity even in new ART regimens (Margolis et al., 2014).

Current guidelines associate two different NRTIs with other antiretroviral families such as integrase inhibitors or, alternatively, with protease inhibitors (PI), which have also been associated with metabolic alterations (Mallon et al., 2005; Domingo et al., 2010; Hammond et al., 2010). To control these subclinical events, a glucose, lipid, and hepatic profile is usually monitored in clinical settings to manage chronic HIV-infected and treated patients aiming to avoid further clinical manifestations (AIDSinfo, 2018).

Although ART has dramatically reduced acquired immune deficiency syndrome (AIDS) development, major concerns have been ascribed to its mitochondrial and metabolic toxicity, especially primary ART (Martinez et al., 2001; Garrabou et al., 2009; Hargreaves et al., 2016). Despite current available drugs and regimens are almost free from toxicity, some of these primary antiretrovirals, including AZT, are still used in certain geographic or clinical settings (World Health Organization, 2018). Both mitochondrial and metabolic disturbances caused by the virus and its ART were postulated as one of the bigger etiological bases of adverse events including hyperlactatemia, hepatic failure, decreased bone mineral density, neuropathy, myopathy, lipodystrophy, and metabolic syndrome (Brinkman et al., 1999; Carr and Cooper, 2000; Pfeffer et al., 2009; Caron-Debarle et al., 2010; Hammond et al., 2010; Güerri-Fernández et al., 2018). However, the contribution of each one of these entities (the virus or its treatment) to associated adverse clinical manifestations is difficult to elucidate in HIV-infected and treated patients. While viral consequences without therapeutic interference have been historically evaluated in naïve patients (Miró et al., 2004), assessment of isolated ART toxicity without viral interference usually requires *in vitro* assays (Kakuda, 2000). Therefore, the *in vivo* consequences for ART for mitochondrial and metabolic toxicity in an HIV-free environment requires novel experimental approaches and cohorts of patients that have been scarcely evaluated to date.

Despite the main goal of ART being the treatment of HIV infection, these drugs may also be used to prevent vertical mother-to-child transmission or can also be administered as pre-exposure or post-exposure prophylaxis (PrEP or PEP, respectively, Yap et al., 2019). PEP involves counseling, assessment of risk of exposure to the infection, HIV testing, and the prescription of a 1-month course of antiretroviral drugs with appropriate support and follow-up (Katz and Gerberding, 1997; Chauveau et al., 2019). While the necessity of PEP is undeniable, it is still limited by a low-adherence, non-negligible secondary effect and some tolerability issues of unknown etiology (Beymer et al., 2017; Chauveau et al., 2019), showing worse tolerability than the ART prescribed for long-term HIV-infected patients under chronic treatment (Rabaud et al., 2005). Such diversity of secondary effects and differential level of mitotoxicity has been attributed to different PEP regimens depending on their composition, but there is little molecular data supporting such differential safety/toxic profile (Groener et al., 2011).

This toxicity has prompted clinical organizations to gradually change the composition of PEP regimens. Between 2008 (Ibarguren et al., 2008) and 2014 (Azkune et al., 2011; World Health Organization, 2013), the PEP regimen consisting of PI plus AZT + 3TC was replaced by a new regimen containing PI plus TDF + FTC. This change in PEP policies offered the perfect occasion to compare these two regimens, which still rank as first-choice treatments in certain patients' cohorts or countries (Supplementary Table 1).

Due to HIV prevalence, the use of PEP is highly advisable when an acknowledged risk of HIV transmission is detected, and there is the need for understanding the secondary or toxic effects of this treatment. PEP-treated patients offer an outstanding opportunity to determine the short-term mitochondrial and metabolic effects of PEP *in vivo*, without viral interference. Hence, we designed the present study to assess whether the 28-day PEP regimens can cause clinical, mitochondrial, or metabolic toxicity and whether there are any variances between the different PEP regimens, thus confirming the usefulness of mitochondrial toxicogenomics for antiretroviral management.

## Materials and Methods

### Design, Criteria, and Participants

We performed a multicentric observational study in HIV-1-exposed and uninfected patients to evaluate mitochondrial and metabolic disturbances before and after a 28-day PEP treatment comparing two different regimens: PI plus AZT + 3TC (*n* = 9) or PI plus TDF + FTC (*n* = 14).

Patients were recruited in two hospitals: the Hospital Clinic of Barcelona (Barcelona, Spain) and the Hospital of Granollers (Granollers, Spain).

All participants initiated their PEP regimen within 48 h after a non-occupational sexual exposure to HIV and provided informed consent to be enrolled in the study, which was approved by the Ethical Committee of our institutions.

The inclusion criteria were adults over 18 years old with no clinical evidence of primary mitochondrial disease, or concomitant treatment with potential toxic drugs for mitochondria (antipsychotics, statins or antibiotics, among others) and the full completion of the 28-day treatment (*per-protocol* analysis).

Although the initial sample of the study included a total of 30 participants, 7 of them were lost or excluded from the study. These excluded participants requiered changes of their PEP regimen due to the manifestation of intolerability recorded during the clinical interview.

Epidemiological, virological, and therapeutic characteristics of the HIV-exposed participants were equivalent in both PEP arms. There were no statistically significant differences between both groups with respect to gender and age distribution. These treatment groups were composed by men exclusively, with mean age ranging from 33 to 34 years. The duration of treatment was consistent in both groups, as all patients received full-length PEP regimen and, once concluded, all participants were negative for HIV antibody testing.

### Epidemiological, Clinical, and Metabolic Data

As aforementioned, epidemiological, virological, and therapeutic parameters including age, gender, HIV antibody (ELISA), PEP regimen, and treatment intervention were gathered during the study. Similarly, data regarding tolerability, adherence, and reasons for PEP discontinuation were collected in the follow-up on account of clinical interviews.

Glucose, lipid, and hepatic profile data included information about blood glucose (measured using the glucose-oxidase method), triglycerides, and total cholesterol (by enzymatic approaches), as well as aspartate and alanine aminotransferase hepatic enzymes (AST and ALT), which were quantified by atomic absorption spectrophotometry (Siemens Diagnostics®, New York).

### Collection of Blood Samples

Fasting samples of 20 ml of venous blood were collected in Vacutainer™ EDTA tubes. For each subject of the study (and for both groups), two sets of samples were obtained, one just after HIV exposure and before PEP, and another after a 28-day course of treatment. Blood was first centrifuged at room temperature for 15 min at 1,500g to reduce platelet contamination through plasma removal. Peripheral blood mononuclear cells (PBMCs) were immediately isolated by means of Ficoll density gradient centrifugation procedure (Histopaque®-1077, Sigma Diagnostics, St. Louis, MO) (Cossarizza, 2003; Mallone et al., 2011). After isolation, PBMCs were resuspended in phosphate-buffered saline and stored frozen at −80°C until analysis.

### Nucleic Acid Isolation From PBMC and Quantification of mtDNA

An aliquot of PBMC was used for extracting total DNA using a standard phenol-chloroform procedure. For mtDNA quantification, a fragment of the mitochondrial conserved gene mt12SrRNA and the nuclear constitutive gene nRNAseP were amplified simultaneously and in duplicate by multiplex quantitative Real-Time PCR. We used Applied Biosystems technology (CA, USA) in a 96-well plate and results were expressed in relative units as the ratio between mtDNA to nuclear DNA (mt12SrRNA/nRNaseP), as previously validated (Côté et al., 2011) and reported by our group (Moren et al., 2015; Catalán-García et al., 2016; Barroso et al., 2019) and other groups (Villarroya et al., 2011; Navarro-Sastre et al., 2012; Carreño-Gago et al., 2019).

### Statistical Analysis

Results were expressed as mean ± standard error of the mean (SEM) or in percentage of change ± SEM with respect to the baseline measurement. Longitudinal differences between both study time points (in each treatment arm) and cross-sectional differences between treatment intervention groups (PI plus AZT + 3TC vs. PI plus TDF + FTC) were determined with the non-parametric Kolmogorov–Smirnov test for paired and independent measures, respectively. Correlation analysis between all quantitative parameters was determined using the non-parametric Spearman test. Statistical analysis was performed using the Statistical Package for Social Sciences version 23.0 (SPSS, Chicago, Illinois, USA). Statistical significance was set at a *p* < 0.05.

## Results

As previously stated, from the initial 30 participants of the study, 7 discontinued PEP before 4 weeks due to gastrointestinal secondary effects including bloating, diarrhea, nausea, and/or vomiting. Consequently, longitudinal mitochondrial and metabolic toxicity profile could not be assessed in these 7 patients due to lack of follow-up. From these patients, 6 were treated with PI plus either AZT + 3TC and 1 with a PI plus TDF + FTC (relative risk or RR for PI plus AZT + 3TC vs. PI plus TDF + FTC discontinuation = 9.33; 95% CI = 1.34–65.23).

After 6 months of HIV exposure, all subjects that continued the study (*n* = 23) remained uninfected and blood analysis for HIV antibodies were all confirmed as negative.

**Figure 1:**
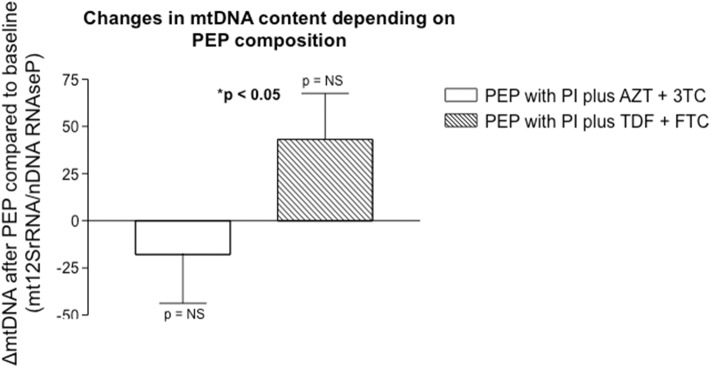
Non significant differences in mtDNA content were observed within each therapeutic group intervention (before and after each treatment), but significant differences were found between the different PEP regimens (PI plus AZT + 3TC vs. PI plus TDF + FTC). Results were expressed as the ratio of mitochondrial 12SrRNA gene with respect to the constitutive nuclear RNAseP gene.

There were no statistically significant intragroup differences between initial and final mtDNA levels within each PEP regimen: baseline 133.5 ± 19.8 mtDNA/nDNA copies vs. final 115.7 ± 22.4 levels for PI plus AZT + 3TC regimen and initial 136.5 ± 20.9 vs. final 177.3 ± 22.8 copies for PI plus TDF + FTC regimen. However, when comparing differences between groups, mtDNA content was significantly reduced in the PI plus AZT + 3TC regimen vs. the PI plus TDF + FTC group: −17.9 ± 25.8% vs. 43.2 ± 24.3%, respectively, *p* < 0.05 (Figure 1).

There were no statistically significant differences before and after treatment in glucose, lipid, or hepatic metabolic profiles in both groups, either concerning glucose, triglycerides, total cholesterol, AST, or ALT levels, regardless of the PEP regimen followed, as summarized in Table 1 and Supplementary Figures 1, 2.

**Table 1:** Glucose, lipid, and hepatic profile of all participants before and after PEP treatment.

HIV-exposed patients					
	**PEP regimen with PI plus AZT** **+** **3TC (*****n*** **=** **9)**		**PEP regimen with PI plus TDF** **+** **FTC (*****n*** **=** **14)**		
	**Before**	**After**	**Before**	**After**	***P*****-value**
Glucose (mg/dl)	93.4 ± 5.4	86.5 ± 3.8	79.5 ± 3.2	94.6 ± 10.0	NS
Triglycerides (mg/dl)	98.5 ± 24.5	106.5 ± 20.8	135.9 ± 19.0	122.6 ± 22.0	NS
Total cholesterol (mg/dl)	181.5 ± 15.9	180.3 ± 17.3	155.3 ± 6.7	181.3 ± 16.4	NS
AST (U/L)	31.4 ± 3.1	30.9 ± 3.6	20.7 ± 1.4	21.6 ± 2.3	NS
ALT (U/L)	29.4 ± 4.8	32.0 ± 5.4	19.7 ± 1.1	27.9 ± 4.7	NS

*No differences were observed in metabolic parameters after the therapeutic intervention or between regimens. All values are expressed as mean ± SEM. ALT, alanine transaminase; AST, aspartate transaminase; AZT, Zidovudine; FTC, Emtricitabine; HIV, human immunodeficiency virus; NS, non-significant; PEP, post-exposure prophylaxis; PI, protease inhibitor; SEM, standard error mean; TDF, Tenofovir; 3TC, Lamivudine*.

**Figure 2:**
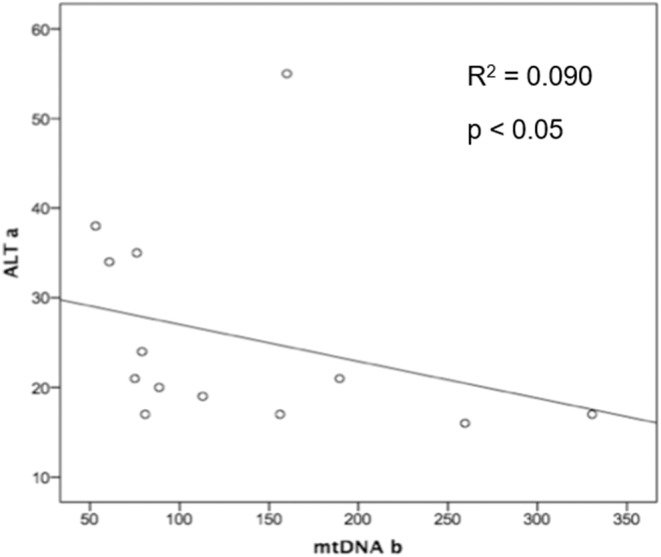
Spearman Rho coefficient was significant and showed a negative correlation for basal levels of ALT and mitochondrial DNA after treatment intervention in both PEP groups (PI plus AZT + 3TC or PI plus TDF + FTC), suggesting that proper basal hepatic function protects from further drug toxicity (*p*-value = 0.015). ALT a, Alanine transaminase baseline levels; AZT, Zidovudine; mtDNA b, mitochondrial DNA after treatment; PEP, post-exposure prophylaxis.

Some metabolic parameters were correlated, showing their strong dependence to maintain physiologic homeostasis (Supplementary Table 2). In addition, mtDNA levels after treatment were negatively correlated to initial ALT levels (*R*^2^ = 0.090 and *p* < 0.05) regardless of the PEP regimen (Figure 2).

## Discussion

HIV infection and ART toxicity (especially of NRTIs) have been postulated as the etiopathological basis of several side effects in HIV-infected and chronically treated patients (Carr and Cooper, 2000; Kohler and Lewis, 2007). Both have been demonstrated to induce mtDNA depletion and derived mitochondrial and metabolic dysfunction (Garrabou et al., 2009; Margolis et al., 2014) even after short periods of treatment (Carr, 2000; Pilon et al., 2002). However, the differential contribution of each agent (HIV or ART) to the observed mitochondrial toxicogenomic profile that is present in HIV-infected patients under ART is difficult to elucidate. Isolated HIV-induced mitochondrial damage has been studied in HIV-infected and untreated individuals (naïve), but ART-related mitochondrial toxicity has been poorly explored in uninfected subjects on account of ethical concerns.

HIV-exposed patients subjected to PEP prophylaxis convey a unique opportunity to test ART toxicity without HIV interference. Additionally, we took advantage of the use of different PEP regimens to compare different clinical, metabolic, and mitochondrial ART toxicity profiles.

Regarding PEP efficacy, all tested alternative treatments showed identical immunotherapeutic efficacy in preventing HIV infection, both in the present study and in the literature (Sultan et al., 2014).

Regarding clinical manifestations and despite its short length (28 days according to up-to-date guidelines), serious complications were raised: the low compliance, the appearance of several secondary or toxic effects, and the little commitment of some patients led to further discontinuation of AZT-containing regimens, herein demonstrated. As previous reported, the main secondary effects for both of these regimens that led to discontinuation were gastrointestinal symptoms (Chowta et al., 2018). These clinical side effects make PEP prone to become a difficult treatment to be fully completed. However, few toxicological studies have been done to assess molecular causes of differential safety/toxic profile of PEP regimens or antiretroviral toxicity in human subjects without HIV interference.

With respect to mitochondrial toxicity, a previous study performed in 18 individuals reported a decrease in the mitochondrial transmembrane potential over a 4 weeks of HIV-PEP, suggesting that PEP toxicity may be confirmed in larger cohorts (Groener et al., 2011). We herein tested the mitochondrial target of nucleoside analog toxicity, considered the gold standard for monitorization of antiretroviral toxicity, that is mtDNA content.

According to our findings, when comparing PEP regimens including PI plus AZT + 3TC with respect PI plus TDF + FTC, subclinical mtDNA depletion was higher in those receiving AZT + 3TC. This confirms previous reported higher mitochondrial toxicity for these older drugs derived from *in vitro* (Kakuda, 2000) or *ex vivo* studies in HIV-infected and long-term treated individuals (Gardner et al., 2013; Sun et al., 2014).

Despite that the use of pyrimidine analogs in PEP regimens, and particularly AZT, is being reduced in developed countries, it still ranks as the alternative option in the CDC, WHO, and EACS guidelines for certain patients (Centers for Disease Control Prevention, 2016; World Health Organization, 2018). Specifically, (i) it is the alternative treatment in subjects over 13 years old with renal dysfunction (creatinine clearance ≤ 59 ml/min); (ii) it is the alternative treatment for children aged 2–12 years; or (iii) it is the preferred treatment for children aging 4 weeks to 2 years old; and (iv) it is the alternative choice of treatment in adults (Battegary et al., 2018). In these cases, AZT is chosen with 3TC. Furthermore, in numerous developing countries, AZT administration in PEP regimens is still the treatment of choice.

These results, among others (Morén et al., 2012; Margolis et al., 2014), give light to the capacity for antiretrovirals to target and disrupt mtDNA expression even after short treatments. Translating all these findings into emerging fields such as epigenetics opens new gates in research to elucidate whether these changes into gene expression can cause drug resistance, metabolic disturbances, and different secondary effects that can lead to drug discontinuance and its subsequent treatment failure (Nyce et al., 1993; Lucarelli et al., 1996; Bozzi et al., 2008; Koczor et al., 2015). It has been shown that some miRNAs that participate in the regulation of mitochondrial translation are mitochondrial-genome-encoded miRNAs (Stimpfel et al., 2018). Consequently, mtDNA depletion produced by NRTIs, as AZT by itself, may reduce miRNA content, thus having effects in mitoepigenetics (Koczor et al., 2015). Additionally, some studies propose a possible surrogate effect in neonates under AZT-containing regimens, as they show an altered nuclear heterochromatin organization that persisted after the treatment was terminated (up to 9 years of age) (Senda et al., 2007; Zuena et al., 2013; García-Otero et al., 2019). Whether all these levels of regulation of mtDNA expression are additionally influencing the toxicity of tested PEP regimens in our work should be addressed in further studies.

Finally, the metabolic profile of PEP-treated patients did not show any differences either in basal or endpoint levels between groups, indicating that in a 28-day interval, there are no visible effects on glucose, lipid, or hepatic enzyme levels regardless of PEP composition. Interestingly, lower initial ALT levels have been associated with higher content in mtDNA after PEP in both groups. While all patients had standard liver enzyme levels, these results point out the association between mitochondrial toxicity and hepatic function, probably because proper basal liver function protects from further drug toxicity by promoting hepatic drug detoxification.

Noticeably, this study has several constraints. The most relevant limitation may be its small sample size. Because of the singularity of these individuals, the lack of compliance, and the need for fast sample processing (to immediately isolate fresh PBMC), it was difficult to gather all the participants for the study in a short period of time. In fact, we needed to perform a multicenter study to include the minimum sample size required to reach our aim. However, we cannot discard a type II error due to the small sample size of the cohorts herein tested, which may be bypassed in further studies with bigger sample sizes and controlled designs. Additionally, the fact that male patients exclusively composed our sample may be considered as the second limitation of the study. However, in current clinical settings, this characteristic may reflect the differences in prevalence of HIV infection according to gender in general population and eradicates potential gender interference in observed results. Regarding the source of sample, we acknowledge that mitochondrial parameters may be exacerbated in more energy-dependent tissues than PBMCs. Likewise, we are aware that assessing specific PBMC composition would be of interest to assess potential interference of cell populations in observed findings, as well as preventing platelet contamination (Tin et al., 2016; Sun et al., 2018). However, we should take into consideration that PBMCs have been demonstrated to be a reliable and non-invasive model to perform mitochondrial studies and that is the present gold standard for mitochondrial toxicity evaluation (Garrabou et al., 2009; Moren et al., 2015; Barroso et al., 2019). Additionally, the potential follow-up of patients for an extended period of time over PEP administration and additional measures for evaluation of mitochondrial toxicity or specific cell toxicity profiling may be of interest for further approaches.

## Conclusions

The results herein presented indicate that, first, short-term ART in the absence of HIV infection can induce mitochondrial toxicity and, second, in the context of HIV-PEP, new antiretrovirals regimens including PI plus TDF + FTC show less mtDNA depletion and therefore are less harmful to mitochondria than the old ones with PI plus AZT + 3TC. The latter regimen also showed a higher risk of drug discontinuation due to a lack of tolerance, while capable of maintaining identical therapeutic activity. Whether mitochondrial toxicity relies at the base of adverse PEP effects has to be further demonstrated. However, considering the reported association between mitochondrial toxicity and clinical adverse effects in chronic antiretrovirals-treated HIV individuals, these results should be considered to elaborate guidelines to potentially reduce tolerability and toxicity issues of PEP.

Fortunately, efforts are being raised to elaborate global policy makers and coordinate program managers, researchers, and activists around the world at a moment of a paradigm shift of the global response to HIV (24), where toxicity of PEP regimens should be considered and AZT should be discouraged.

PEP-treated patients convey an outstanding opportunity to assess antiretroviral toxicity *in vivo* and mtDNA is confirmed as the gold standard for mitochondrial toxicogenomics in antiretroviral management.

## Data Availability Statement

The datasets generated for this study are available on request to the corresponding author.

## Ethics Statement

The studies involving human participants were reviewed and approved by Ethical Committee of Hospital Clinic of Barcelona. The patients/participants provided their written informed consent to participate in this study.

## Author Contributions

We also acknowledge the contribution of each author: GG, along with EM, FC, EP, and ÒM, who conceived the study and supervised the data collection and analysis. JR and AL participated in patient inclusion and supervised the clinical aspects of this study, in collaboration with JM and EM. Each author participated in the recruitment of epidemiological, clinical, and metabolic data of patients. GG, MB, MG-M, and IG-C managed the collection of samples and isolation of cells. EL, JC-S, DJ, CM, and MB were responsible for experimental analysis of mitochondrial DNA content, under the supervision of GG. ET provided technical assistance for reagent preparation. A database was also created by MB and SB to collect all the clinical and experimental parameters to perform the statistical analysis of the data, under the supervision of GG. All authors participated in drafting and critical revision of the manuscript, especially MB, SB, EM, and GG.

## Conflict of Interest

The authors declare that the research was conducted in the absence of any commercial or financial relationships that could be construed as a potential conflict of interest.
