# Supplementary material for: Mitochondrial Toxicogenomics for Antiretroviral Management: HIV Post-exposure Prophylaxis in Uninfected Patients
Source: Front Genet. 2020 May 26;11:497. doi: 10.3389/fgene.2020.00497 (PMC7264262; doi:10.3389/fgene.2020.00497)
Supplement: Supplementary file 1 [file Data_Sheet_1.docx]

**Supplementary material**

**Supplementary Table ST1:** Simplification of ARV treatment for PEP according to the new guidelines updates.

| **(Centers for Disease Control and Prevention, 2016)** | | | **(World Health Organization, 2018)** | | | **EACS** (Battegary et al., 2018) | |
| --- | --- | --- | --- | --- | --- | --- | --- |
| **Subjects 13 years old and pregnant women with normal renal function** | Preferred | TDF+FTC  +  RAL or DTG | **Adults including pregnant and breast-feeding** | Preferred | TDF+FTC (or 3TC)  +  DTG | Preferred | TDF+FTC  +  RAL |
|  | Alternative | TDF+FTC  +  DRV+RTV |  | Alternative | TDF+FTC (or 3TC)  +  EFV or PI/r |  |  |
| **Renal Dysfunction (Creatinine clearance 59mL/min)** | Preferred | AZT+3TC  +  RAL or DTG | **Woman willing to become pregnant** | Preferred | TDF+FTC (or 3TC)  +  EFV |  |  |
|  | Alternative | AZT+3TC  +  DRV+RTV |  | Alternative | TDF+FTC (or 3TC)  +  ATV/r or RAL | Alternative | AZT+3TC  +  RAL or DRV |
| **Children aged 2-12 years** | Preferred | TDF+FTC  +  RAL | **Children** | Preferred | ABC + 3TC  +  DTG |  |  |
|  | Alternative | AZT+3TC  +  RAL or LPV/r |  | Alternative | ABC + 3TC  +  LPV or RAL or EFV or NVP |  |  |
| **Children from 4 weeks to 2 years old** | Preferred | AZT+3TC  +  RAL or LPV/r | **Neonates** | Preferred | AZT+3TC  +  RAL | Alternative | TDF+FTC  +  DTG |
|  | Alternative | AZT+FTC  +  RAL or LPV/r |  | Alternative | AZT+3TC  +  NVP or LPV |  |  |

CDC: Center for Disease Control and Prevention; WHO: World Health Organization; EACS: European AIDS Clinical Society. Nucleoside Reverse Transcriptase Inhibitors (NRTIs): TDF – Tenofovir; FTC – Emtricitavine; AZT – Zidovudine; 3TC – Lamivudine; ABC - Abacavir. Non-Nucleoside Reverse Transcriptase Inhibitors (NNRTIs): EFV – Efavirenz; NVP: Nevirapina. Integrase Inhibitors: RAL – Raltegravir; DTG – Dolutegravir; Protease Inhibitors: DRV – Dorunavir; RTV – Ritonavir; LPV – Lopinavir; LPV/r – Lopinavir potentiated with ritonavir; ATV/r: Atazanavir.

**Supplementary Table ST2: Spearman correlation test between mitochondrial and metabolic parameters.** Table shows all the correlations between the different variables included in the study before and after PI+AZT+3TC and PI+TDF+FTC treatment. Significant correlations resulted between: mtDNA before and ALT levels before treatment (R^2^=0.233; p=0.015); mtDNA after and ALT levels before treatment (R^2^=0.090; p=0.042); TAG before and AST levels before treatment (R^2^=0.303; p=0.008); TAG before and AST levels after treatment (R^2^=0.432; p=0.005); TAG before and ALT levels after treatment (R^2^=0.475; p=0.003); TAG after and CHOL levels after treatment (R^2^=0.092; p=0.031); TAG after and ALT levels before treatment (R^2^=0.265; p=0.018); CHOL before and CHOL levels after treatment (R^2^=0.636; p=0.007); CHOL after and AST levels after treatment (R^2^=0.200; p=0.045); AST before and AST levels after treatment (R^2^=0.715; p=0.003); AST before and ALT levels after treatment (R^2^=0.551; p=0.015); AST after and ALT levels after treatment (R^2^=0.534; p=0.002). As most of metabolic parameters are mutually dependent, special emphasis is given to alternative correlations, including those between mitochondrial and metabolic parameters. This is the case of the correlation between mtDNA content and ALT levels, supporting the association between mitochondrial toxicity and secondary effects in the hepatic function (see graphical representation in Figure 2).


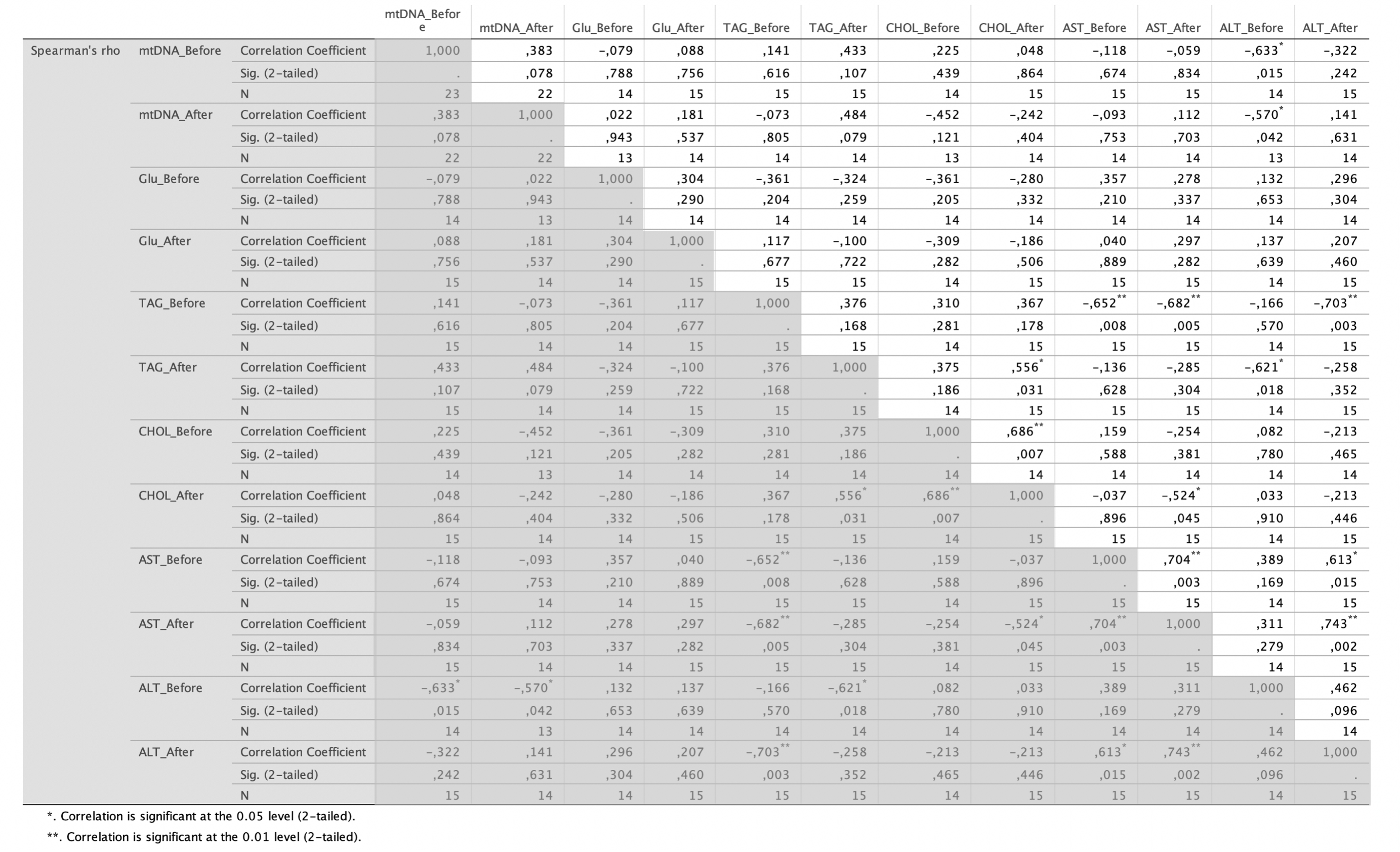


PI, Protease inhibitor; AZT, Zidovudine; 3TC, Lamivudine; TDF, Tenofovir; FTC, Emtricitabine; mtDNA, mitochondrial DNA; GLU, Glucose; TAG, Triglycerides; CHOL, Total Cholesterol; AST, Aspartate transaminase; ALT, Alanine transaminase.

**Supplementary Figure SF1: Graphic representation of mitochondrial and metabolic findings before and after PI+AZT+3TC treatment.** None of them resulted in statistic significant changes. **A)** Glucose; **B)** Triglycerides; **C)** Total cholesterol; **D)** Aspartate transaminase; **E)** Alanine transaminase.

~~
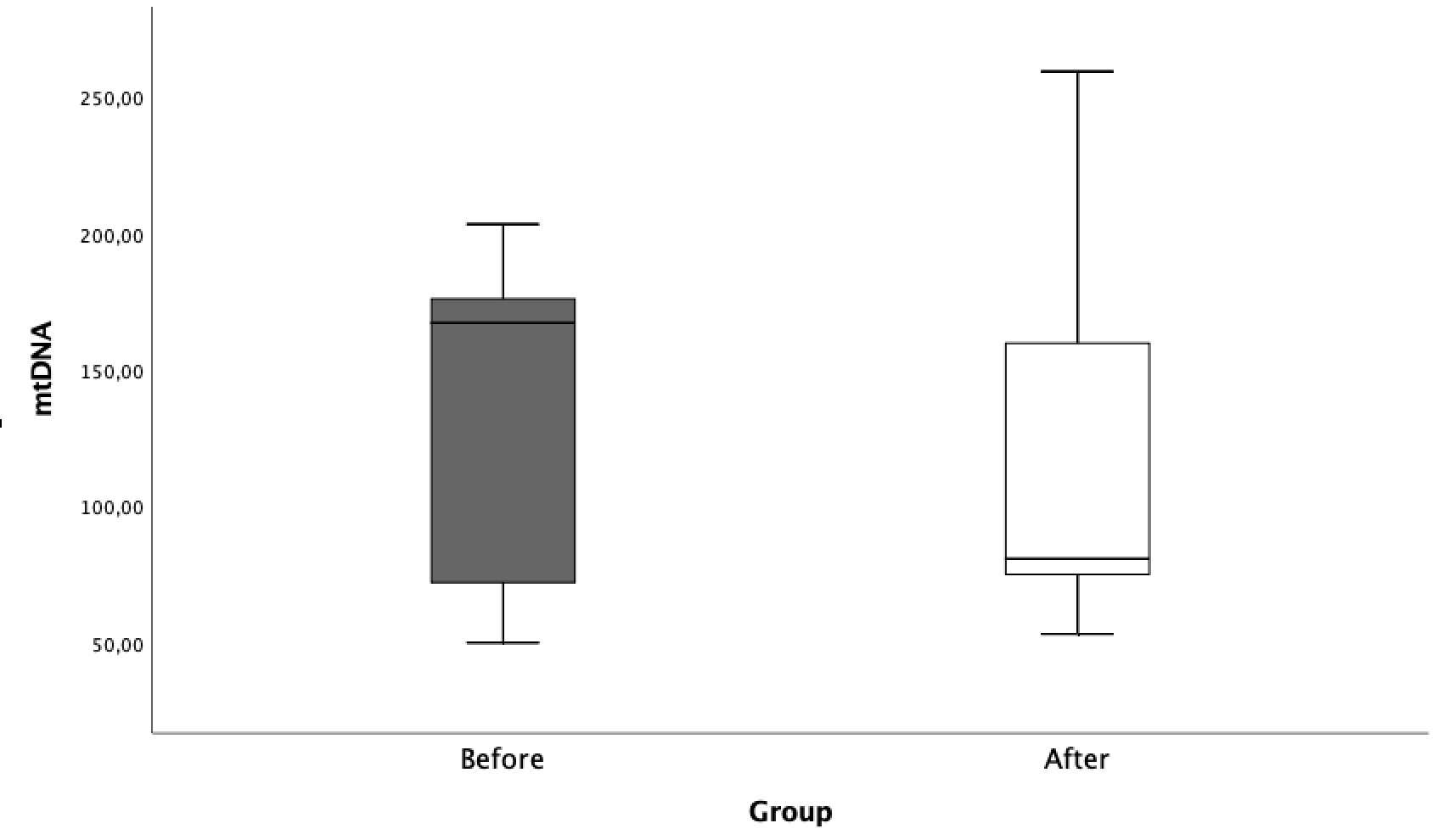

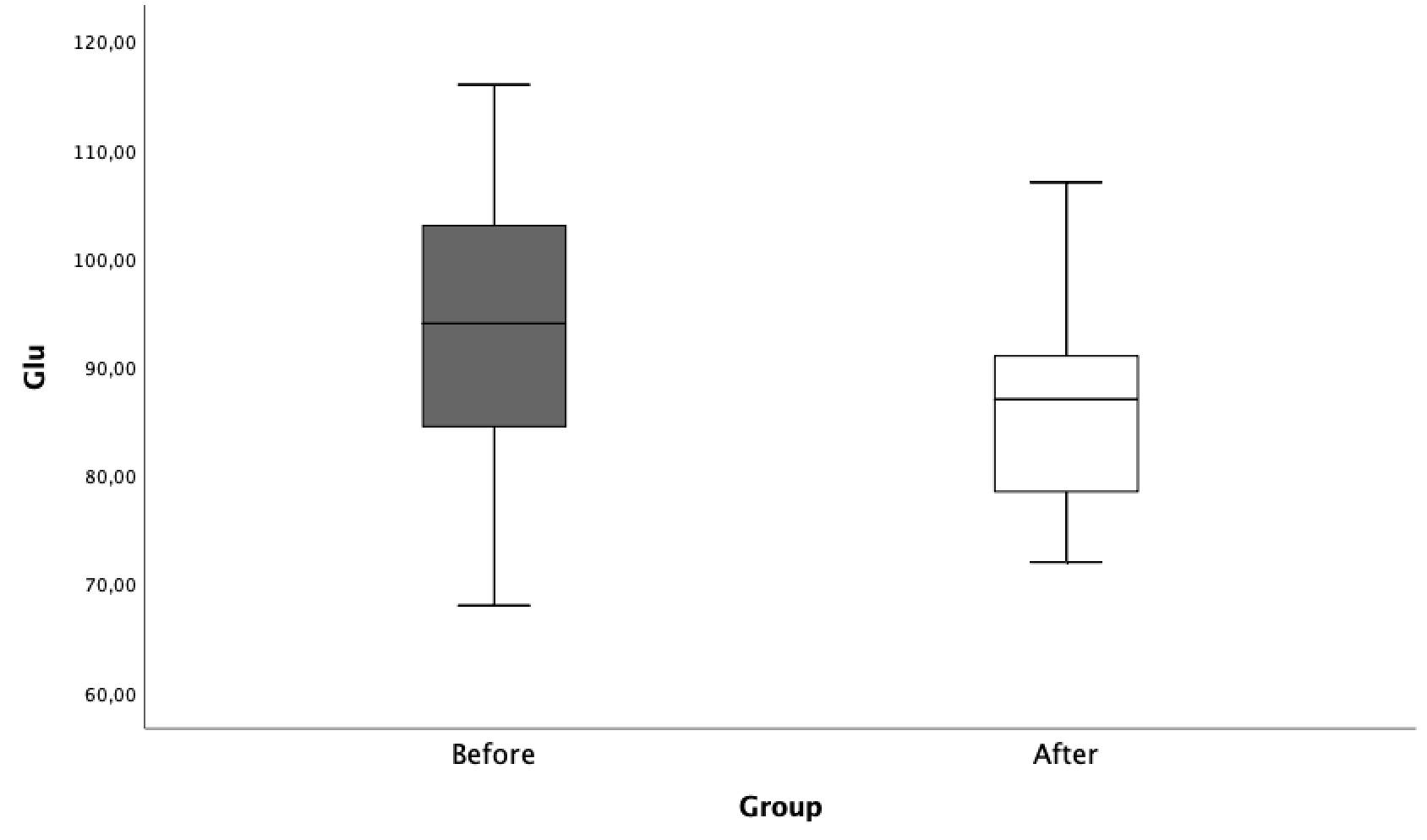
~~

B

A

D

C

~~
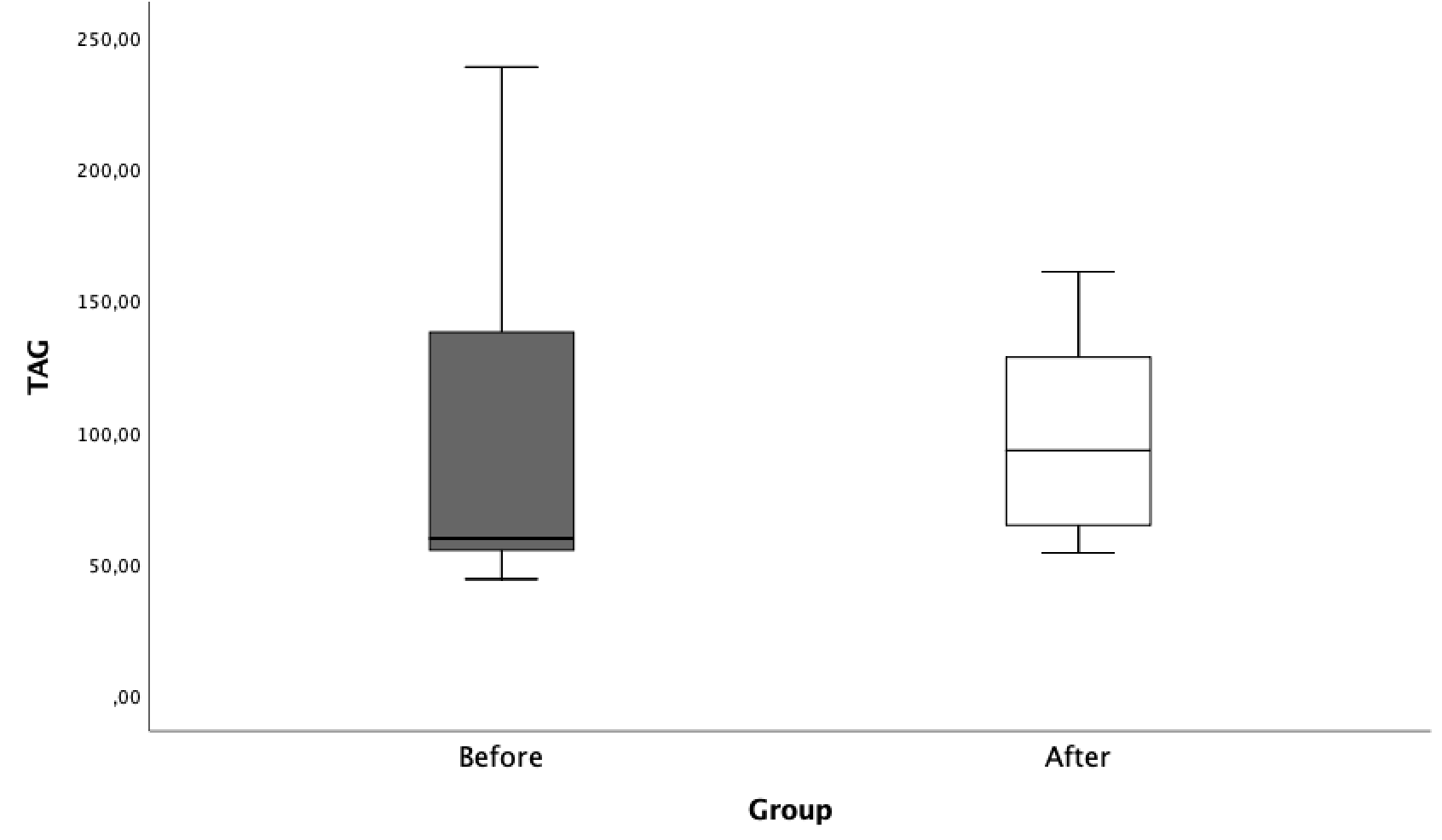

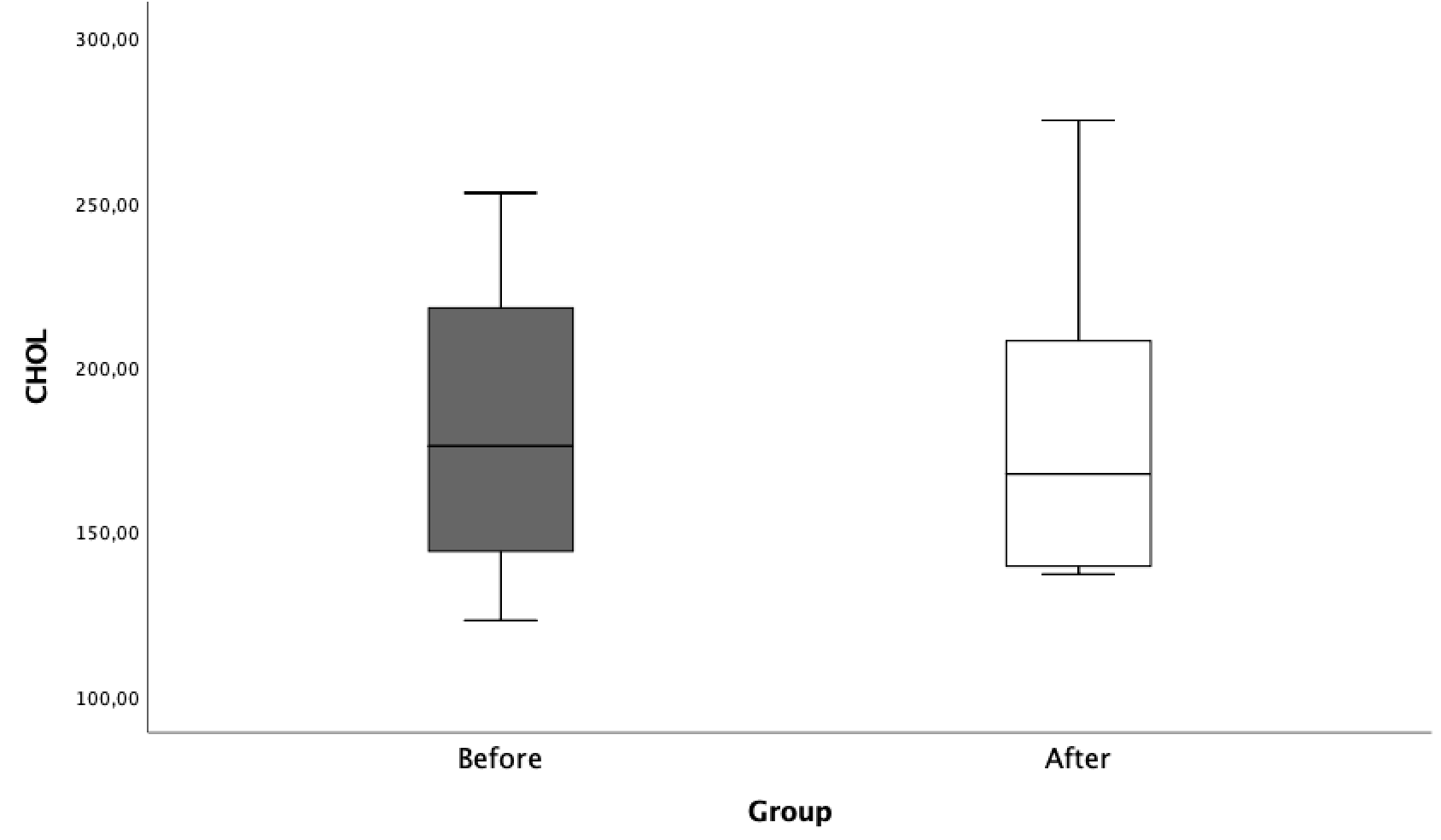
~~

F

E

~~
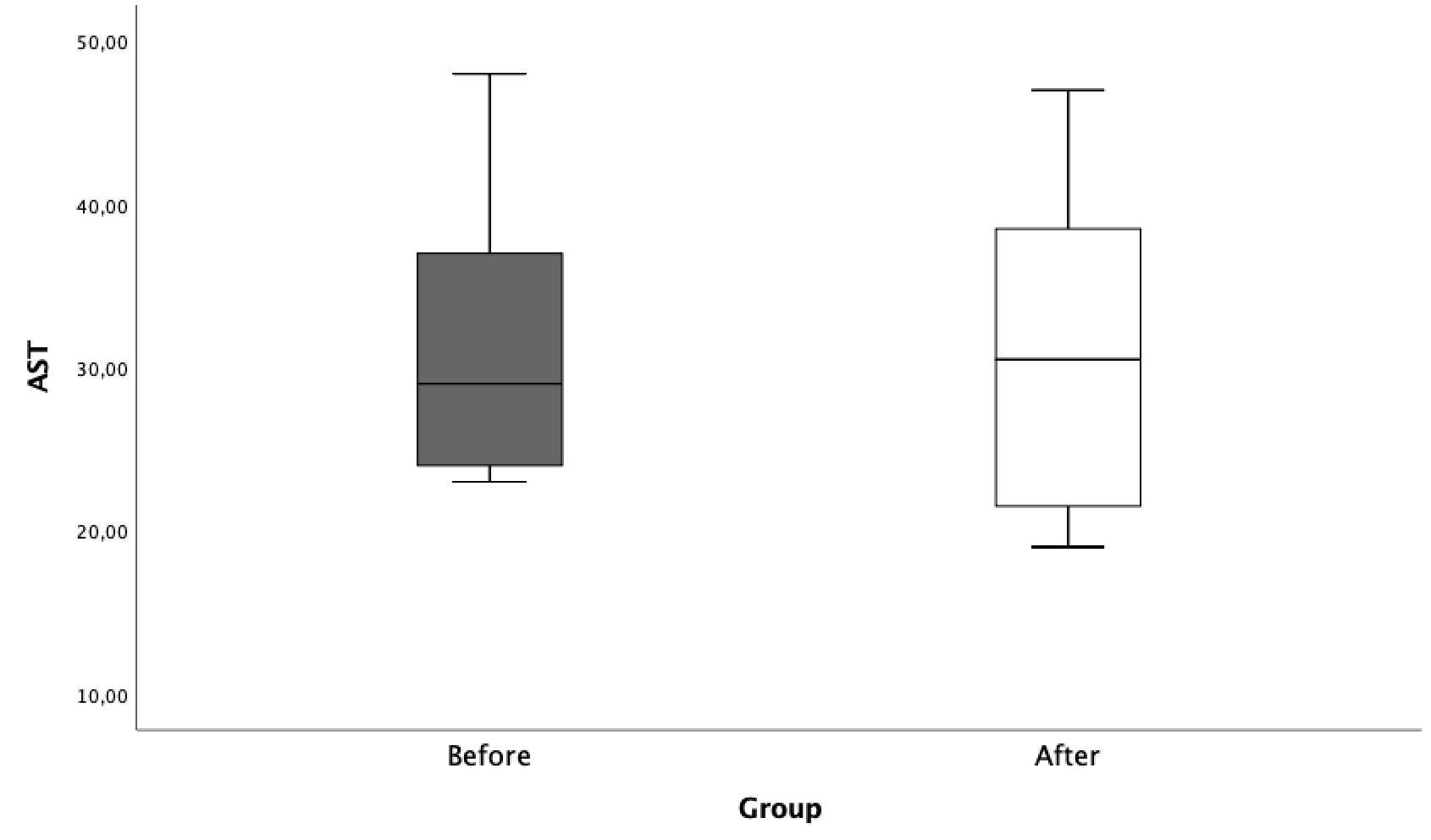

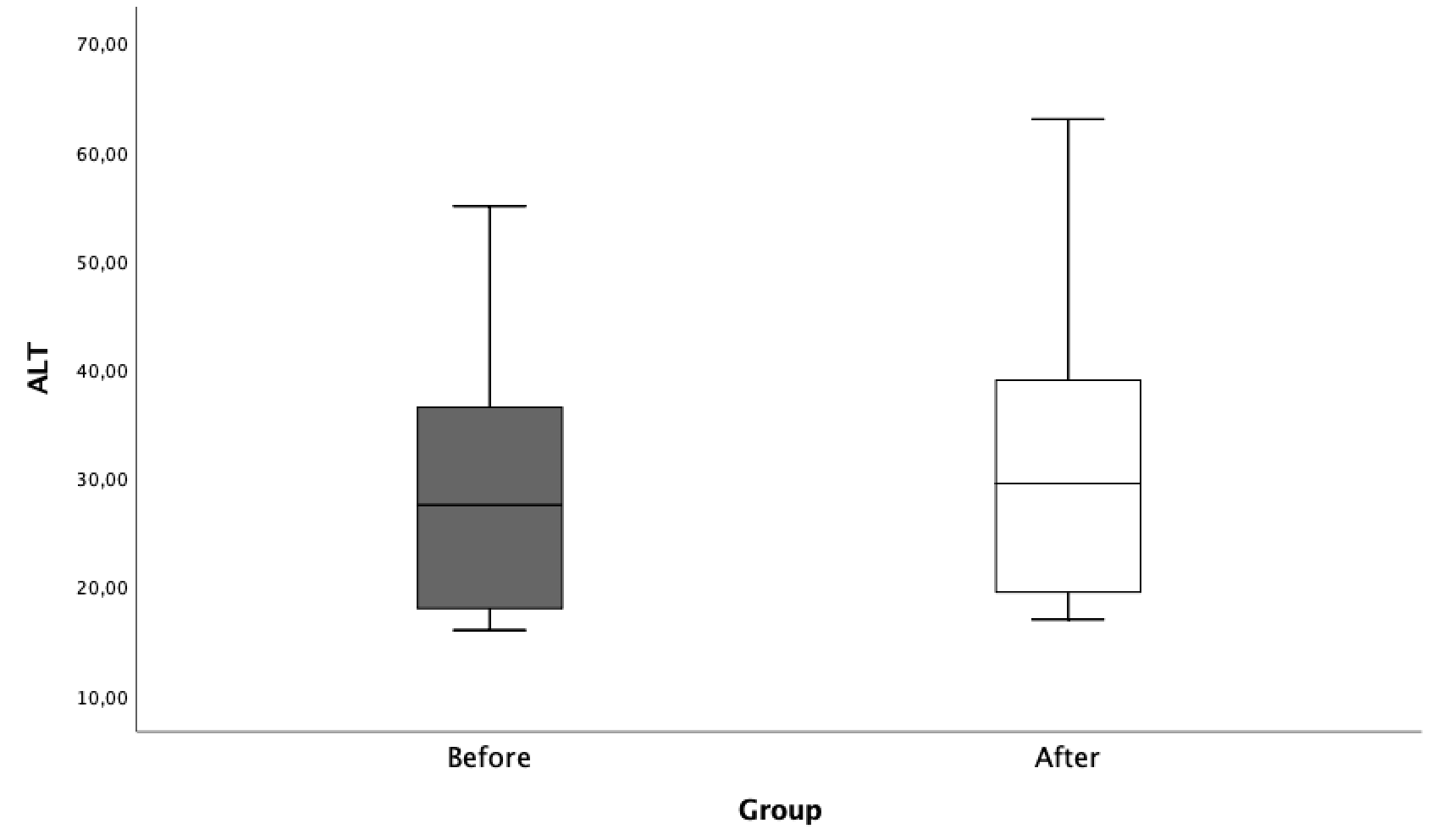
~~

**Supplementary Figure SF2: Graphic representation of mitochondrial and metabolic findings before and after PI+TDF+FTC treatment.** None of them resulted in statistic significant changes. **A)** Glucose; **B)** Triglycerides; **C)** Total cholesterol; **D)** Aspartate transaminase; **E)** Alanine transaminase.

~~
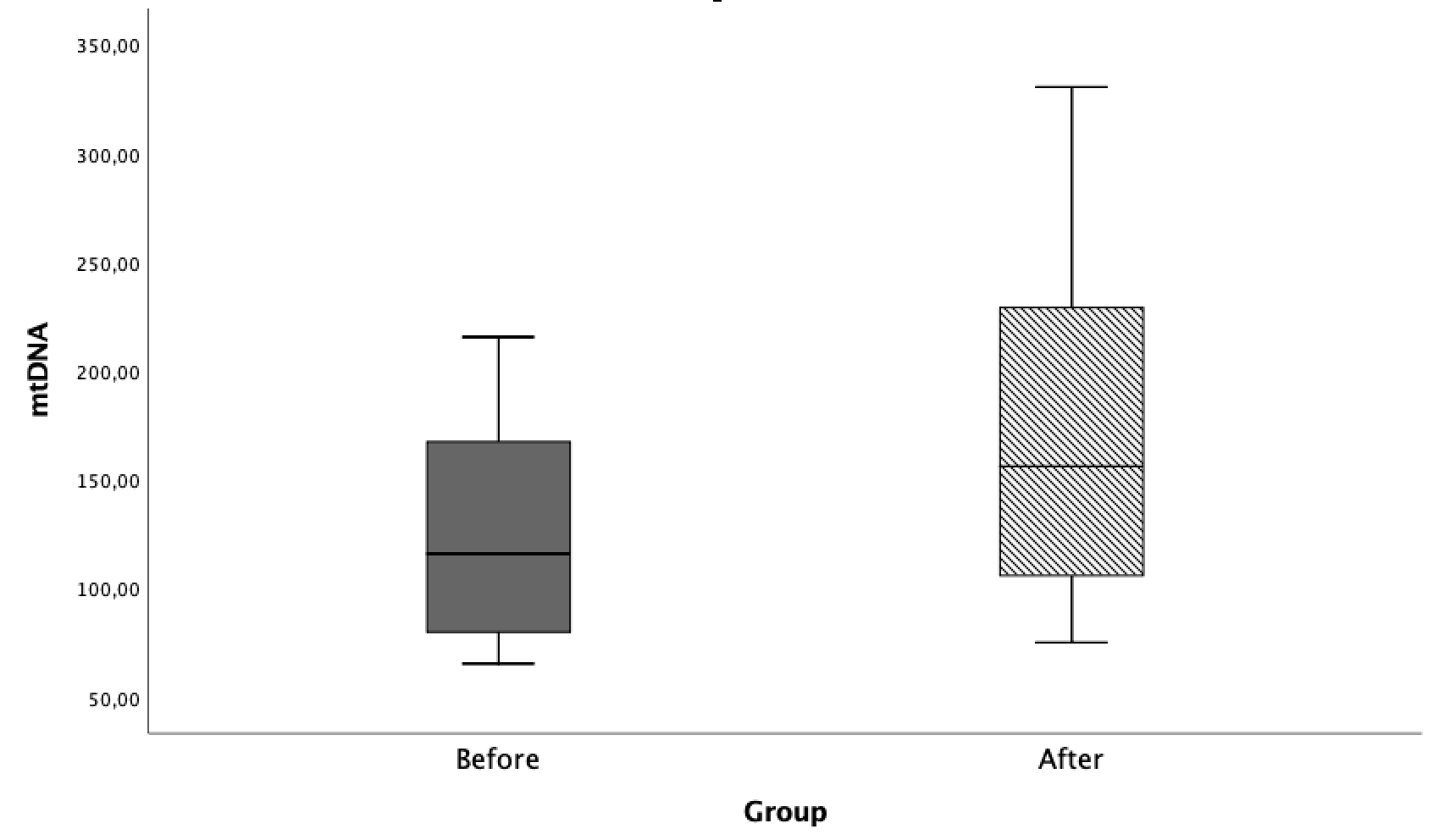

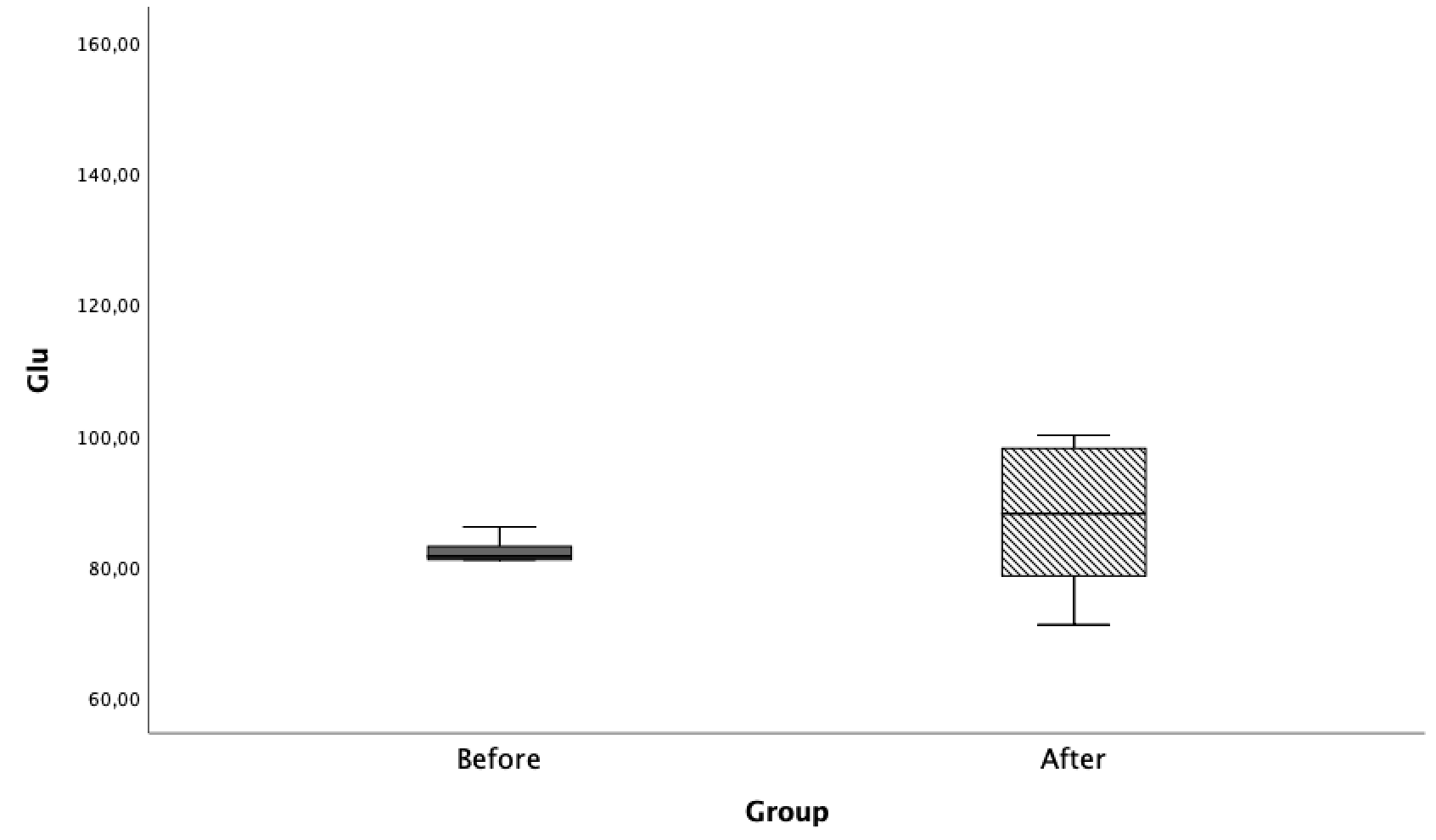
~~

A

B

C

D

E

F

~~
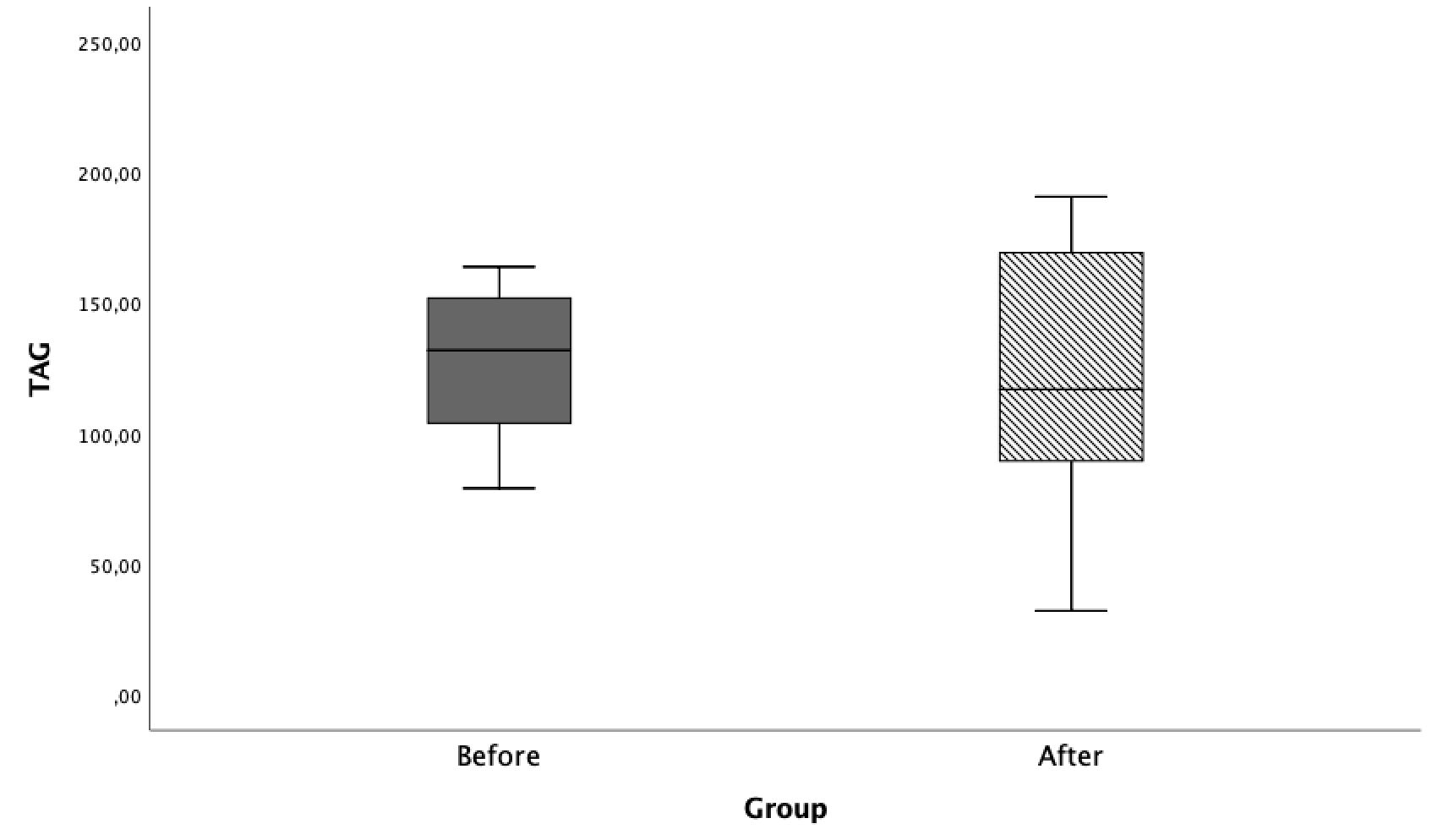

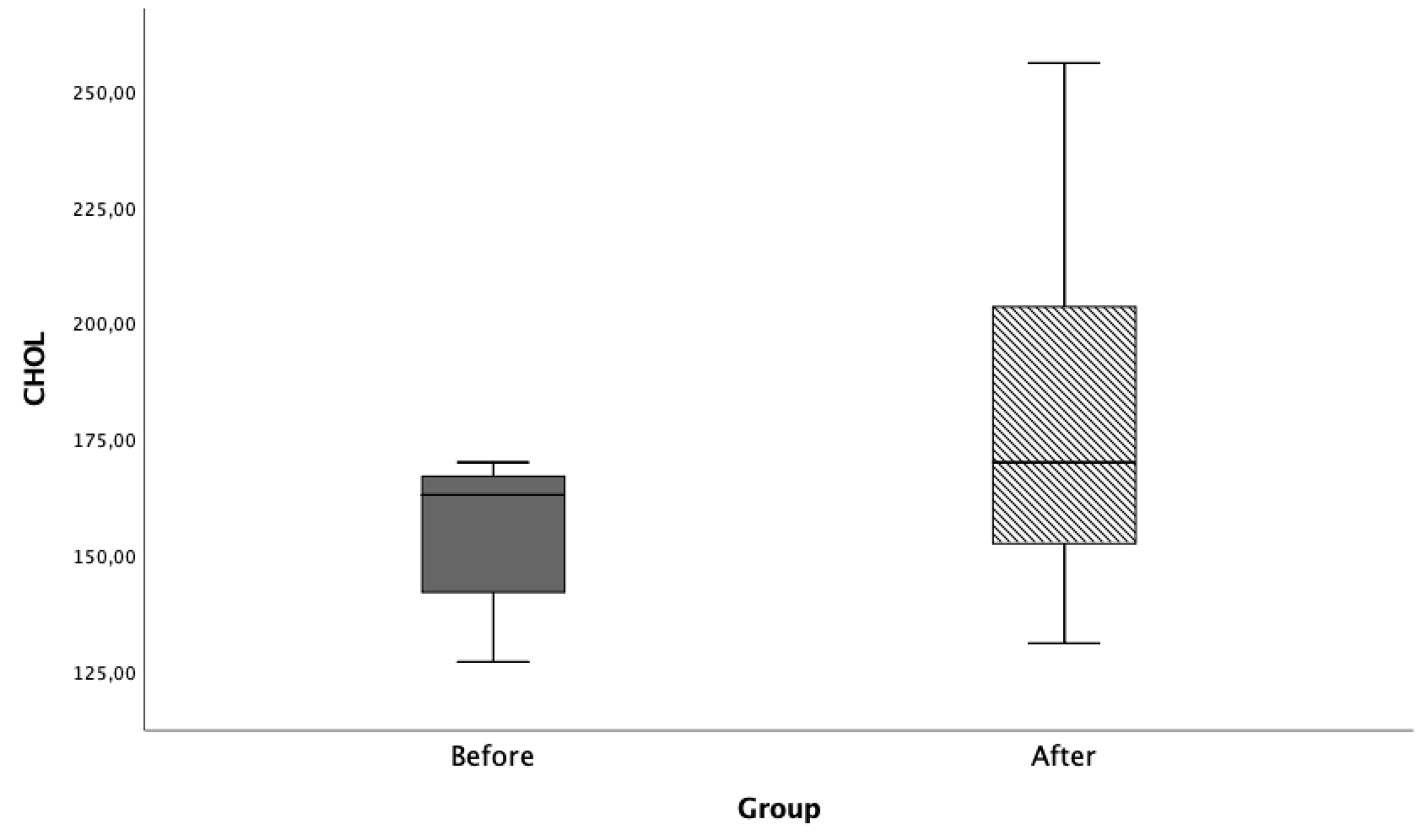
~~

~~
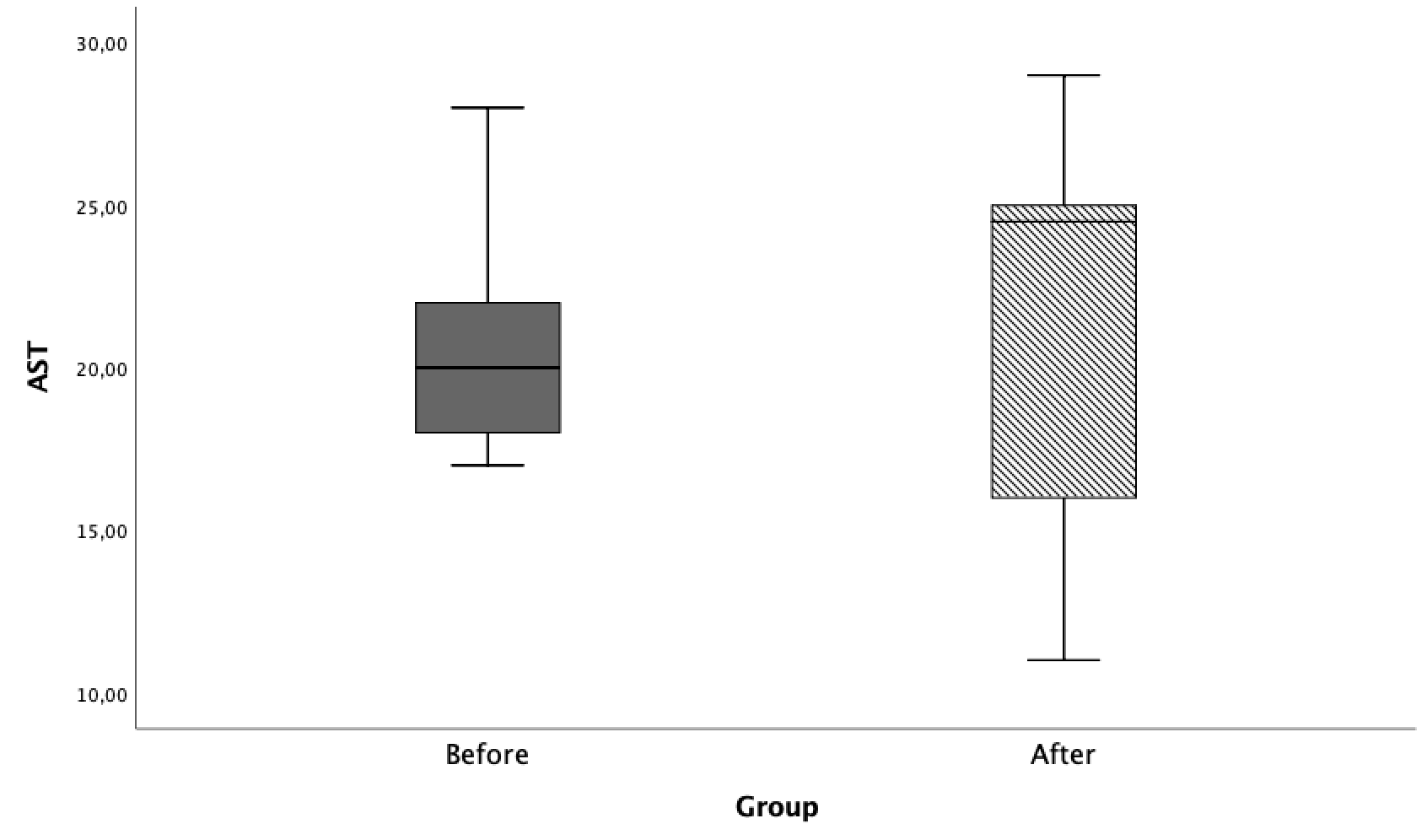

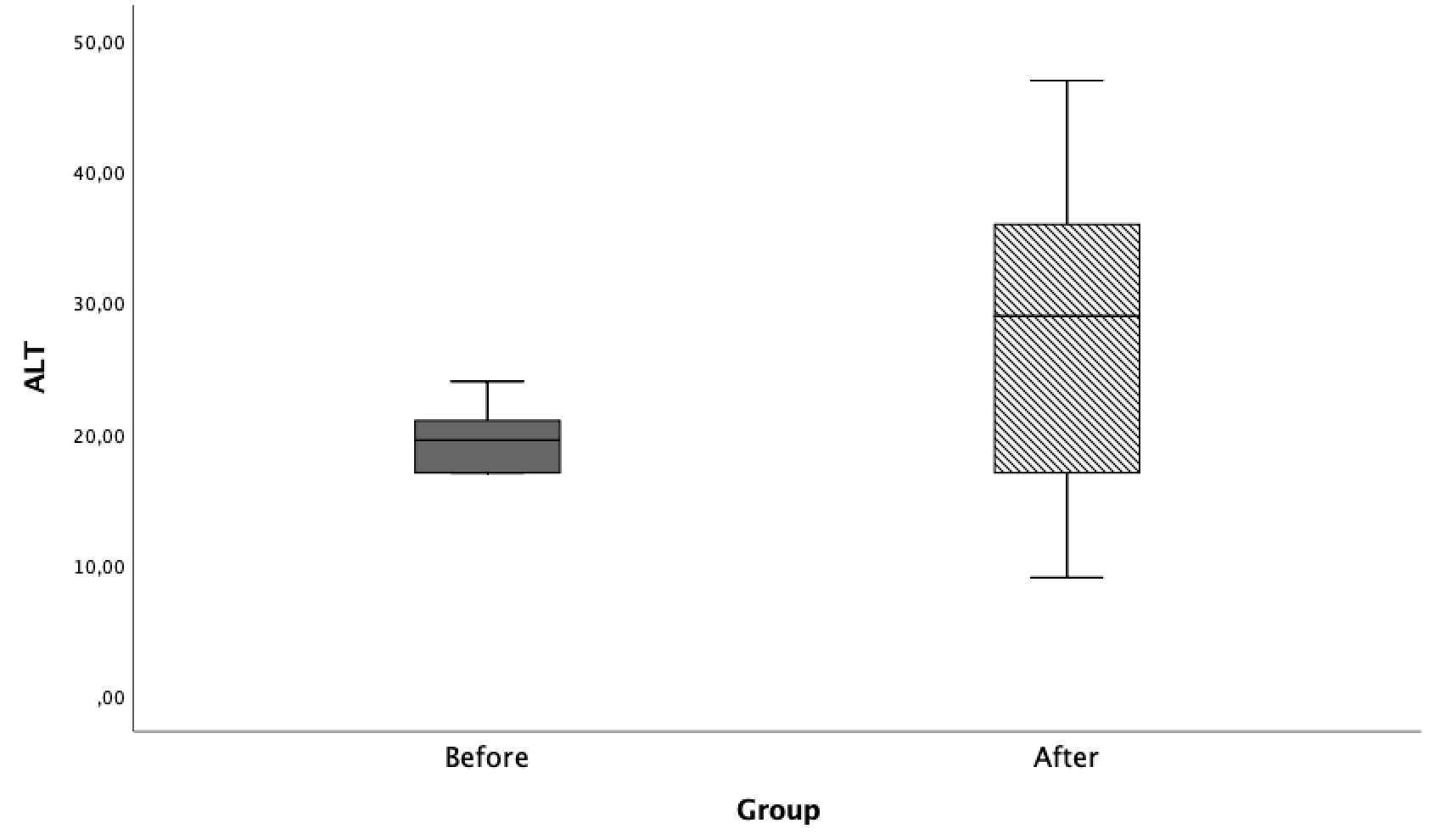
~~
